# Proactivity's hidden ceiling: how organizational conditions undermine the sustainability of proactive behavior

**DOI:** 10.3389/fpsyg.2026.1911096

**Published:** 2026-09-09

**Authors:** Francesco Cangiano

**Affiliations:** 1 Department of Defense and Security, Rabdan Academy, Abu Dhabi, United Arab Emirates; 2 Bond Business School, Bond University, Robina, QLD, Australia

**Keywords:** proactive behavior, proactivity sustainability, self-determination theory, conservation of resources theory, competitive climate, psychological safety, work design

## Abstract

Many organizations invest in developing proactive employees while simultaneously maintaining structural conditions that make proactivity unsustainable. This paper addresses that contradiction. Although research has documented the psychological costs of proactive behavior at the episode level, how these dynamics accumulate into longer-term behavioral trajectories remains theoretically underdeveloped, as does the role of organizational conditions in shaping which trajectory employees follow. Integrating Conservation of Resources theory with Self-Determination Theory, a multilevel framework is developed that identifies four proactivity trajectories over time: sustainable, contingent, declining, and suppressed, tracked jointly through an employee's visible initiative and the self-efficacy and motivational quality supporting it. Organizational structural conditions are identified as predictors of trajectory type, setting a “ceiling” within which individual regulatory resources operate. Performance management and leadership behavior translate these conditions into daily employee experience. Eleven propositions and a future research agenda are derived. The framework argues that sustaining proactivity over time is primarily an organizational design problem, not an individual development one, with direct implications for HR practice, work design, and the management of initiative at work.

## Introduction

Proactive work behavior, self-initiated, future-oriented action aimed at changing oneself or one's work environment (Parker et al., 2006), has attracted sustained scholarly attention. Organizations actively promote it, and for good reason: proactivity predicts individual performance (Grant et al., 2009), team productivity (Hyatt and Ruddy, 1997), and firm success (Frese and Fay, 2001). Contemporary work contexts have only intensified this demand. As organizations operate in increasingly complex, uncertain, and rapidly changing environments, the premium placed on employee initiative has grown correspondingly (Bindl and Parker, 2011; Crant, 2000).

The dominant framing in the literature has been antecedent-focused: identifying who is proactive, when they are proactive, and what motivates proactivity (Grant and Ashford, 2008; Parker et al., 2010; Xu et al., 2026). A smaller but theoretically important literature has turned to consequences, examining the conditions under which proactivity is resource-generating or resource-depleting (e.g., Bolino et al., 2010; Cangiano and Parker, 2016; Fay and Hüttges, 2017; Strauss et al., 2017). This work has produced two important advances. First, proactivity's wellbeing consequences are not uniform, they depend on contingencies including motivational quality, feedback, job control, and climate (Cangiano et al., 2019; Strauss et al., 2017; Wehrt and Sonnentag, 2024). Second, at the within-person level, the episode-level resource consequences of proactivity depend on the balance between resource investment and perceived resource return: when proactive efforts are appraised as effective, resource gains offset investment costs; when appraised as ineffective, the same investment produces net depletion (Cangiano et al., 2026).

What the literature has not done, however, is explain how episode-level dynamics accumulate into longer-term behavioral trajectories, or how organizational-level conditions shape which trajectory an employee follows. The field's predominant short-window diary designs are well-suited to capturing episode dynamics but unable to observe longer term phenomena (Ohly et al., 2010). Thus, we understand the daily mechanics of proactive resource dynamics with increasing precision but cannot yet explain why some employees sustain proactivity over months and years while others progressively withdraw, and what organizations can do about it.

This paper addresses that gap. Many organizational contexts, as currently designed, contain structural features that work against the sustained proactivity they simultaneously encourage. The reward structures, performance management practices, competitive climates, and work design features that characterize many contemporary organizations can progressively deplete the psychological conditions under which proactivity sustains itself. This structural constraint is what the present paper terms the *hidden ceiling*: a structural limit on how far individual resources can sustain initiative over time, regardless of individual motivation or capacity. Proactivity sustainability refers to the extent to which employees can continue initiating future-oriented change over time without progressive resource depletion, motivational drift, or withdrawal. Motivational drift refers to the gradual shift in the basis of proactive behavior from autonomous toward increasingly controlled regulation across repeated episodes. This is distinct from what motivates individual proactive episodes, which has been the primary focus of the antecedent literature (Parker et al., 2010; Strauss and Parker, 2014). It is also distinct from questions about the effectiveness or outcomes of particular proactive acts. Proactivity sustainability is a longitudinal construct concerning the trajectory of proactive behavior over weeks, months, and years, and its determinants are partly individual and partly organizational.

The trajectories developed below track a specific subset of proactive behaviors: socially visible, change-oriented proactive acts that seek to alter work methods, procedures, or organizational practices (Parker and Collins, 2010). Personal initiative, taking charge, and voice are relevant here only when they fall within this domain. Job crafting, which can often be pursued inconspicuously and without needing anyone's approval, falls outside this domain; it appears later in the framework only as an organizational condition, job crafting latitude, that shapes how safely and effectively employees can pursue the tracked forms of proactivity, not as a tracked outcome in its own right. Defining the domain this way does not make it homogeneous. An employee may sustain voice while withdrawing from taking charge, or the reverse, since these behaviors carry different levels of visibility and interpersonal risk even inside the change-oriented domain. The trajectories developed here describe an employee's overall pattern within the domain. Where behaviors inside it move independently, the assumption of one trajectory per employee holds only approximately.

To develop this argument, a multilevel framework is proposed that integrates three components: episode-level resource balance mechanisms, rooted in Conservation of Resources (COR) theory (Hobfoll, 1989; Hobfoll et al., 2018); a typology of four proactivity trajectories that describe how episode-level dynamics accumulate over time; and a set of organizational-level structural conditions, work design and job crafting latitude, reward systems and competitive climate, psychological safety, and employment insecurity, that influence how far individual regulatory resources can carry proactive behavior over time. A set of testable propositions and a multilevel research agenda is then derived. The paper concludes with a discussion of implications for the management of proactivity in AI-augmented work environments, where new pressures complicate the already fragile conditions for sustainable initiative.

**Figure 1:**
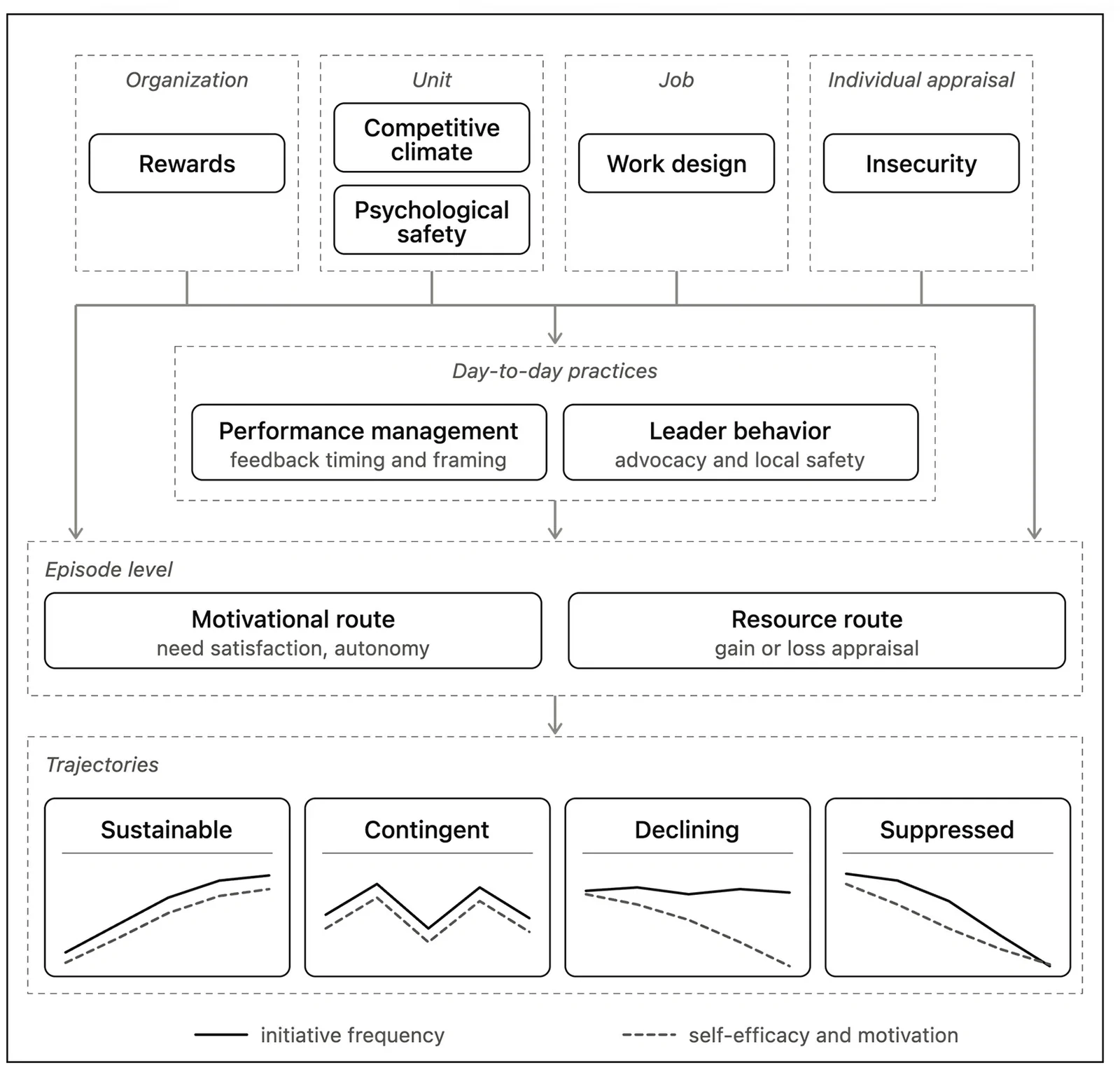
Multilevel model of organizational conditions and proactivity trajectories. Dashed containers mark the level of analysis at which each element operates. Organizational conditions reach the episode-level mechanisms both directly and through two day-to-day practices. Each trajectory panel plots initiative frequency (solid line) and role breadth self-efficacy and motivational quality (dashed line) over time; the declining trajectory is the case in which the two diverge.

Figure 1 shows the chain from organizational conditions to the episode-level mechanisms and on to the trajectory outcomes. Conditions operate both directly and through the two day-to-day practices, and each is marked with the level at which it operates. Each element is developed in the sections that follow.

This is a theoretical paper. It builds a multilevel framework of how organizational conditions shape whether proactive behavior can be sustained over time, and derives propositions for empirical work to test. It makes three key contributions. Theoretically, the framework extends the proactivity literature from episode-level contingencies to longitudinal trajectory dynamics, and from individual-level moderators to organizational structural conditions as drivers of sustainability, generating testable propositions to drive future research on the topic. From a practical perspective, it shifts attention from developing proactive individuals to designing organizations in which proactive behavior can be sustained. This matters now more than it did a decade ago. AI integration may shift the proactive initiatives left to humans toward precisely those that are most emotionally costly and hardest to evaluate. Precarious employment pushes employees to perform initiative for visibility instead of enacting it from genuine motivation. And rising performance demands mean that the structural conditions most likely to deplete proactive behavior (e.g., internal competition, outcome-focused feedback) are also the ones organizations under pressure tend to resort to. A framework that identifies these conditions and specifies how they undermine proactivity is therefore both theoretically necessary and practically relevant.

## Theoretical foundations

COR theory holds that individuals are motivated to acquire, maintain, and protect resources, defined broadly as objects, conditions, personal characteristics, and energies that hold value either instrumentally or in themselves (Hobfoll, 1989; Hobfoll and Shirom, 2001). It consists of three foundational principles. First, resource loss is disproportionately more salient and impactful than equivalent resource gain, a principle Hobfoll et al. (2018) term loss primacy. Second, those who lack resources are more vulnerable to further loss, and initial losses tend to beget additional losses, creating loss spirals. Third, resource investment is a strategy for protecting against loss and achieving gain, but individuals remain especially vulnerable when investments fail to yield expected returns (Halbesleben et al., 2014). Applied to proactivity, COR provides a framework for understanding why the same behavior can be resource-generating in some conditions and resource-depleting in others. Proactive behavior constitutes a form of resource investment, it consumes self-regulatory resources with the expectation of future gains in the form of mastery, competence, autonomy, or environmental improvement (Bindl et al., 2012; Hobfoll et al., 2018). Whether this investment pays off depends on the balance between resources expended and resources returned, and on how individuals appraise that balance (Halbesleben et al., 2014; Hobfoll et al., 2018). Proactive behavior refers to self-initiated, future-oriented action taken to change oneself or one's work environment (Parker et al., 2006), encompassing behaviors as varied as taking charge, voice, personal initiative, and job crafting (Bindl and Parker, 2011; Parker and Collins, 2010). Parker et al. (2010) identified three motivational states that prompt and sustain proactivity (i.e., can-do, reason-to, and energized-to), and established that the autonomous vs. controlled basis of motivation is a central moderator of its consequences (Strauss and Parker, 2014; Strauss et al., 2017).

### The costs of proactivity

Proactive behavior is demanding. It requires employees to act without instruction, often against existing arrangements, with no guarantee that the effort will be recognized or that it will work. This costs cognitively, emotionally, and motivationally, and the costs tend to accumulate (Bolino et al., 2010). The evidence, so far, supports this view: daily proactivity predicts elevated cortisol and reduced wellbeing (Fay and Hüttges, 2017), can spill over into difficulty switching off after work (Cangiano et al., 2021), and when resources run low, leads to withdrawal rather than continued initiative (Pingel et al., 2019). Whether these costs materialize depends on several factors. For example, proactivity is more likely to produce strain when motivation is controlled (Strauss et al., 2017), when autonomy and support are limited (Zacher et al., 2019), and when the self-control demands of taking charge are not offset by sufficient job control (Wehrt and Sonnentag, 2024). Cangiano et al. (2026) found that proactivity predicted lower end-of-day depletion when employees felt their efforts had made a difference, suggesting that perceived effectiveness of one's proactive behavior can offset the associated investment cost. But this only held in less competitive workplaces. In highly competitive climates, even successful proactivity yielded weaker psychological returns, because competitive contexts make people evaluate outcomes through social comparison (Fletcher et al., 2008; Hobfoll et al., 2018). What this line of research has not addressed is how these daily dynamics accumulate, whether a pattern of effective proactivity builds on itself over time, or whether repeated depletion wears down the capacity and motivation to keep initiating at all.

### From episodes to trajectories

Short-window diary designs cannot capture trajectory-level phenomena (Ohly et al., 2010), and organizational variables have been treated primarily as contextual moderators rather than as independent determinants of whether proactivity remains viable over time. The present paper argues that organizational conditions do not merely moderate individual-level effects, they set the limit within which individual resources operate. A note on theoretical positioning is necessary before developing the framework. Strauss and Parker (2014) examined effective and sustained proactivity from a self-determination theory perspective, arguing that autonomous motivation is the key condition for proactivity that is both sustained and non-depleting. The present paper builds on that insight but departs from it in two ways. First, where Strauss and Parker (2014) asked what motivates sustained proactivity at the individual level, this paper asks how repeated proactive episodes accumulate into distinct trajectory classes over time. The present framework also runs on two mechanisms rather than one, and both are carried explicitly. The first is motivational. Conditions that satisfy basic psychological needs sustain autonomous motivation, and autonomously motivated initiative is less depleting to enact (Ryan and Deci, 2000; Strauss et al., 2017). The second is resource-based. Each proactive episode ends in net gain or net loss depending on how its return is appraised, and those balances accumulate across episodes (Hobfoll et al., 2018). Strauss and Parker (2014) developed the first. The contribution here is to run both together while keeping them distinct, since motivational quality and resource balance often move together and can come apart. The work-design, competition, and insecurity propositions each specify predictions along both routes. More importantly, autonomous motivation is necessary but not sufficient: it cannot sustain proactivity indefinitely in organizations that chronically attenuate gain appraisals, shift initiative toward controlled regulation, or elevate the emotional costs of proactive risk-taking. This paper examines why the organizational conditions employees most commonly work under tend to undermine sustained proactive behavior over time.

## Proactivity trajectories over time

Whether resources build or wear down across repeated episodes shapes the long-term behavioral pattern an employee follows. COR theory's loss spiral logic (Hobfoll, 1989; Hobfoll et al., 2018) helps explain why episodes ending in net resource loss lower the base available for the next proactive investment, increasing the cost threshold of future episodes and reducing the probability that subsequent episodes will achieve net gain. Conversely, episodes ending in net gain create resource surpluses that facilitate subsequent investment, what Hobfoll et al. (2018) describe as resource caravans, in which resources accumulate together and reinforce one another. Four empirically distinguishable trajectories emerge from this accumulation logic, which are elaborated below in detail. The four classes are theoretically derived from these accumulation mechanisms; they are not extracted from an existing empirical taxonomy of proactivity trajectories, which does not yet exist. Adjacent literatures nonetheless make heterogeneous classes empirically plausible: person-oriented longitudinal studies of burnout routinely recover qualitatively distinct developmental trajectories, stable, increasing, decreasing, and curvilinear, rather than a single average trend (Mäkikangas and Kinnunen, 2016). Because visible initiative and the self-efficacy and motivation behind it can move independently, as the declining trajectory below illustrates, each trajectory is tracked on both: how often someone initiates, and whether their confidence and motivation to keep doing so are holding up. The two usually move together. The second track bundles two things: an employee's confidence that they can act beyond their core role, and the quality of the motivation behind acting. The framework treats them as one process because, under most conditions, the same experiences move both. When repeated initiatives are appraised as ineffective, confidence and motivation erode together; when they are appraised as effective, both are sustained. Proposition 3 below specifies the one condition where the two should come apart: chronic appraisals of ineffectiveness undermine confidence while the motivation behind acting stays autonomous. Separation between the two outside that condition would count against treating them as one process. When they diverge, someone can keep initiating at the same rate while slowly running out of the confidence and motivation that make it worth continuing. Decline, once it starts, is unlikely to be steady: each costly episode lowers the resources available for the next one and pushes motivation from autonomous toward controlled, which is itself more draining, so losses should build on each other rather than add up at a fixed rate. Two parameters of these trajectories are left unspecified here. The typology does not fix the point at which a contingent pattern becomes a declining one, and it does not establish whether decline can be reversed once the motivational basis has weakened. Both are empirical questions the framework raises without answering, and both are taken up in the research agenda below.

### Sustainable proactivity

In the sustainable trajectory, effectiveness appraisals are consistently sufficient to generate episode-level resource gains that offset investment costs. The motivational basis of proactivity remains autonomous, enacted because it is intrinsically satisfying or personally meaningful, and basic psychological need satisfaction is maintained across episodes (Ryan and Deci, 2000; Strauss and Parker, 2014). A history of effective proactivity builds role breadth self-efficacy, an employee's confidence in carrying out tasks that reach beyond the prescribed technical core of the job (Parker, 1998). Role breadth self-efficacy is treated throughout this paper as part of what the trajectories track, and it does not enter the framework as a separate antecedent of proactive behavior. It is nonetheless dynamic: an employee's between-person level of role breadth self-efficacy forms part of the starting resource base, whereas within-person change across episodes is an outcome of prior proactive episodes that then shapes the cost and perceived feasibility of later ones. Empirically, these correspond to the distinction between initial level and change over time. As it accumulates it reduces perceived social risk, lowering the emotional cost of future episodes. This trajectory is self-reinforcing, sustainable proactivity builds the individual and contextual conditions that make continued proactivity sustainable. However, sustainable proactivity does not rise without limit: gains level off as they approach what the surrounding conditions can support, and the trajectory settles there rather than climbing indefinitely. Longitudinal research complicates this picture: Zacher et al. (2019) found that increases in personal initiative predicted increases in job autonomy, but also detrimental indirect effects on engagement and exhaustion through mood. This suggests that even when proactivity changes the work environment in potentially resource-building ways, its wellbeing consequences depend on whether the broader context allows those gains to translate into sustained psychological resources.

### Contingent proactivity

In the contingent trajectory, episodes vary in effectiveness, producing alternating resource gain and loss. Workers remain proactive but self-regulate quantity and intensity in response to resource levels, increasing proactivity when resources are sufficient and pulling back when depleted. Sonnentag (2003) demonstrated that day-level recovery during off-work time was associated with increased proactive behavior the following day, suggesting that individuals adjust proactive investment depending on their available resources. This trajectory is resource-preserving at the individual level but suboptimal at the organizational level: the regulation is driven by resource constraint rather than genuine choice, and the ceiling on proactivity is set by available resources instead of genuine motivation or opportunity.

### Declining proactivity

In the declining trajectory, repeated episodes of ineffective proactivity, or effective proactivity that goes unrecognized or is enacted under competitive pressure, trigger progressive resource depletion. As resources diminish, the motivational basis of proactivity shifts from autonomous toward controlled regulation; not a deliberate choice but a psychological response to chronic resource loss and frustrated need satisfaction (Ryan and Deci, 2000; Strauss et al., 2017). An employee may continue to exhibit proactive behavior, showing surface-level initiative to meet organizational expectations or protect their position, but the behavior is increasingly controlled and, thus, depleting. Röllmann et al. (2021) found that job insecurity did shape the fatigue consequences of voice over time: voice predicted increases in fatigue when job insecurity was high, but decreases in fatigue when job insecurity was low. These findings suggest that insecure employment conditions do not necessarily eliminate the energizing benefits of voice, but they can activate its resource-depleting consequences. Building on this, when proactive work behavior leads to resource depletion rather than gain, the result is employee withdrawal (Pingel et al., 2019), the behavioral outcome of this trajectory. This trajectory carries substantial burnout risk (Demerouti et al., 2001) and is organizationally damaging because the proactivity it produces is disconnected from genuine change orientation. An employee in a high competitive climate who repeatedly takes initiative, sees the credit claimed by a more politically skilled colleague, and receives outcome-focused feedback that their efforts produced no measurable result, will continue to exhibit surface-level initiative (e.g., attending the right meetings, making suggestions in public), while possibly choosing not to invest genuine cognitive and emotional resources. In this trajectory, proactivity is still visible on the surface, but its motivational and resource basis is deteriorating.

### Suppressed proactivity

In the suppressed trajectory, proactive behavior is withdrawn. Role breadth self-efficacy has declined over time. The individual withdraws from proactive engagement, either overtly or through learned passivity, focusing on core tasks while ceasing to initiate beyond core work requirements. An employee who spent months taking initiative in a low psychological safety climate may initially persist despite punitive, dismissive, or indifferent responses. Over time, however, these responses may signal that proactive effort is unlikely to be welcomed or effective at all. As role breadth self-efficacy fades, the employee withholds proactive behavior and may stop conceiving proactive goals in the first place. Instead of seeing problems as opportunities for change, the employee comes to interpret them as outside their influence or not worth the personal risk of addressing.

*Proposition 1: Repeated net gains in resource balance will hold an employee's initiative frequency and their role breadth self-efficacy and autonomous motivation on a common path, producing a sustainable trajectory where gains are consistent, and a contingent trajectory where they alternate with losses. Repeated net losses will produce one of two patterns. Where initiative frequency is maintained while self-efficacy and autonomous motivation fall, the trajectory is declining. Where both fall together, it is suppressed*.

*Proposition 2: Individual regulatory capacity, autonomous motivation, and dispositional proactivity will predict trajectory only when organizational conditions are not chronically depleting; under sufficiently depleting conditions, those conditions cap what even highly capable employees can sustain and pull them toward decline regardless of their individual resources. Conditions count as chronically depleting when, for a sustained period, the typical proactive episode ends in a net resource loss. How many episodes it takes, and how long the pattern must persist before it hardens into a trajectory, are empirical questions, in line with the parameters left open above*.

There is a condition under which the two routes predict different outcomes. In some organizations the work is meaningful, autonomy is real, and colleagues are supportive, yet initiatives routinely stall in approval processes or disappear without visible effect. Psychological needs are met while effectiveness appraisals stay chronically low. A need-satisfaction account predicts sustained proactivity here: everything it requires is present. The accumulation account predicts decline: each episode ends in a loss appraisal, no matter how autonomously it was motivated.

*Proposition 3: Where organizational conditions support psychological need satisfaction but chronically attenuate effectiveness appraisals, autonomous motivation will be maintained while role breadth self-efficacy declines and initiative frequency eventually follows*.

What an observer would see is the declining trajectory: initiative held at first while confidence erodes underneath it, then falling once confidence can no longer carry it. If the condition persists, the pattern ends in suppression. There are two ways into decline: motivation shifts toward controlled regulation, or, as here, confidence erodes while motivation stays autonomous. The second is what a need-quality account cannot predict. The two routes are therefore specified as parallel mediators, operating together rather than as rival explanations, so a single model can estimate both and test the contrast directly. A weaker version of the same logic appears later in the psychological safety proposition: there, the cost of initiative is paid at the moment of acting, whatever the outcome.

## Organizational conditions for sustainable proactivity

Sustainable proactivity depends not only on whether employees are motivated or capable of being proactive, but on whether organizational conditions allow proactive effort to generate resources over repeated episodes. Four conditions shape this: work design and job crafting latitude, reward structures and competitive climate, psychological safety, and employment insecurity. These elements share a selection criterion: prior theory or evidence links each one directly to how proactive episodes are appraised, through the resource return they yield, the motivational quality behind them, or the social risk they carry. Work design was selected because it bears on both resource return and motivational quality. Consistent with COR and SDT, job autonomy and feedback richness are established antecedents of proactive motivation and behavior (Parker et al., 2006, 2010; Strauss and Parker, 2014), and Zacher et al. (2019) show that autonomy and personal initiative can reinforce one another over time. Reward systems and competitive climate influence both motivational quality and the perceived return on proactive investment: SDT holds that controlling rewards undermine autonomous motivation (Ryan and Deci, 2000), and equity theory holds that competitive contexts lead employees to evaluate the return on their initiative relative to that received by others (Adams, 1965; Cangiano et al., 2026). Psychological safety directly structures the social risk attached to initiative and is a well-established predictor of interpersonally risky behavior such as voice and proactive action (Edmondson, 1999; Edmondson and Lei, 2014). Employment insecurity is a continuing threat to valued resources, consistent with COR logic (Hobfoll et al., 2018), and is associated with reduced capacity for discretionary investment (Greenhalgh and Rosenblatt, 1984; Lebel et al., 2023). Two practices give many of these conditions their day-to-day expression. Performance management is included because formal evaluation systems decide how proactive contributions are assessed and recognized. Immediate leadership is included because leaders control much of what follows an initiative: the feedback it gets, the recognition it earns, and the protection available if (or when) it backfires. The two practices are not the only route, though: the conditions above also bear on proactive episodes directly. Other influences, such as organizational justice and team norms, plausibly work through the same mechanisms; they are extensions of the framework, not conditions judged unimportant. Some of these conditions are formal arrangements anyone can observe; others are perceptions. Either way, their effect on episode-level resource balance runs through how employees experience and appraise the circumstances around a proactive act. This is why employment insecurity, below, is modeled at the level of appraisal.

These conditions sit at different levels. Reward systems are formal, organization-level policy. So are performance management systems, including appraisal cycles and forced distributions, although leaders enact them locally. Competitive climate and psychological safety are unit-level shared climates, properties of the team an employee works in, not of the organization as a whole or of that employee alone (Edmondson, 1999). Work design, including job autonomy and crafting latitude, sits at the level of the job itself. Leadership operates at the level of the individual leader, whose response to a given employee's initiative may differ from the climate the broader unit reports. Employment insecurity has an organizational antecedent, restructuring, layoff risk, but the proposition concerning it is specified at the level of the individual's own appraisal of that threat, since it is the appraisal, not the policy itself, that shapes the resource dynamics described above. Self-efficacy, motivational quality, and the episode-by-episode resource dynamics themselves sit at the level of the individual employee across repeated episodes.

Two amplifying processes explain why organizational conditions produce stronger effects on proactive than on prescribed task behavior. The first is psychological ownership over the work role. Because proactive behavior is self-initiated and directed toward changes the employee has chosen to act on, it engages all three routes through which ownership develops: exercising control over the target, coming to know it intimately, and investing self in it (Pierce et al., 2001, 2003). This amplifies resource dynamics in both directions. When proactive initiatives succeed and are recognized, the ownership dimension magnifies resource gain beyond what competence satisfaction alone would predict. When they fail, are blocked, or go unrecognized, ownership amplifies resource loss: the failure is more ego-threatening than equivalent failure in prescribed tasks because the self was more deeply invested (Pierce et al., 2003). Organizational conditions that suppress recognition therefore tend to produce larger resource losses from proactive episodes than their equivalent effects on prescribed behavior.

The second is need frustration as distinct from need dissatisfaction. Need frustration, the active thwarting of basic psychological needs, produces motivational costs not predicted by the mere absence of need satisfaction (Vansteenkiste and Ryan, 2013). Competitive climates frustrate the need for competence by rendering it comparative. Low psychological safety frustrates the need for relatedness by making social connection contingent on behavioral conformity. Organizations that actively frustrate basic needs push employees toward controlled, depleting forms of proactivity more rapidly than conditions that merely fail to support them. Both amplifying processes operate across all four conditions described below, and are not re-introduced in each subsection.

### Work design and job crafting

Job autonomy and feedback richness are established antecedents of proactive motivation (Parker, 1998; Parker et al., 2006, 2010). High job autonomy lets employees select and shape proactive initiatives in ways that raise the odds of a good outcome, and feedback-rich roles supply the information needed to judge whether an initiative actually worked. Both also build role breadth self-efficacy over time, which reduces the social risk of proactive behavior by making initiative feel within-role. Wehrt and Sonnentag (2024) found that morning taking charge predicted higher end-of-day depletion when job control was low and lower depletion when job control was high, an effect strongest under high self-control demands.

The present framework extends this beyond initiation: work design shapes not just whether people start being proactive, but whether that effort is likely to be experienced as effective, and whether it builds confidence or wears it down over time. These effects accumulate across episodes rather than showing up in any single act, so they should emerge more clearly in longitudinal than cross-sectional designs. Role breadth self-efficacy, as defined above, is one of the things these conditions build or wear down across episodes. It is tracked here as an outcome, not as a predictor. For instance, an employee in a narrowly defined, low-autonomy role who identifies a process improvement faces higher social risk in acting on it, gets less timely feedback on whether it worked, and experiences the initiative as role-transgressive as opposed to role-consistent. Over months, this suppresses effectiveness appraisals and weakens ownership over the change domain, as the role simply gives less back for the same effort regardless of the employee's capability.

Job crafting latitude works the same way, at the point where initiatives get chosen rather than carried out. Job crafting refers to employees' self-initiated efforts to modify aspects of their work, including the tasks they perform, the relationships they build, and how they frame their role (Tims et al., 2012). Latitude to craft matters here because it lets employees direct proactive effort toward what is feasible and likely to be noticed, instead of leaving that choice to chance. What sustains proactivity is the organizational permission to make that choice, not any individual skill at crafting itself. The two are likely to be correlated in practice, but they are distinct: job autonomy is discretion over how existing work is done; job crafting latitude is permission to change what that work is.

*Proposition 4: Job autonomy, feedback richness, and job crafting latitude will each predict sustained proactivity over time through two routes: a resource route, in which they raise the odds that proactive effort is appraised as effective, and a motivational route, in which they support autonomous motivation directly*.

### Rewards, recognition, and competition

Reward structures are a theoretically underexplored determinant of proactivity sustainability. Individual pay contingent on performance, forced-distribution appraisals, and competitive promotion structures create conditions in which proactivity is instrumentally motivated: performed to gain relative advantage or avoid relative disadvantage rather than from genuine change orientation (Appelbaum et al., 2000). Controlled motivation is the primary condition under which proactivity produces strain; when proactivity is autonomously motivated, resource loss is substantially attenuated (Strauss et al., 2017). Competitive reward structures shift the motivational basis of proactivity in a controlled direction, so employees in such systems come to experience their proactive behavior as depleting as opposed to energizing. This is the motivational route.

The resource route runs differently, through how much an employee's investment is seen to be worth. Equity theory holds that people weigh their own input-to-return ratio against that of relevant others, and experience strain when that comparison feels unjust (Adams, 1965): an employee who invests proactively and sees little recognition will appraise that investment as a loss regardless of its objective outcome. Competitive climate compounds this by changing the basis on which the return itself is judged. In high competitive climates, employees assess proactive outcomes not in absolute terms but through comparison with colleagues (Fletcher et al., 2008), so even effective initiative produces a weaker psychological payoff when its value is measured against a colleague's relative position rather than the problem it solved. Forced ranking, competitive promotion structures, and zero-sum credit allocation work through this route: they reduce the effectiveness appraisal and recognition that would otherwise offset the cost of the investment. Cangiano et al. (2026) found that proactivity effectiveness predicted lower end-of-day depletion in low but not high competitive climates, consistent with this mechanism. These effects will not be uniform. Employees high in trait competitiveness may be depleted less by competitive environments, and contingent rewards carry weaker motivational costs when they inform rather than control (Fletcher et al., 2008).

*Proposition 5: Competitive reward systems and high competitive climate will predict steeper proactivity decline over time through two routes: a motivational route, in which competition shifts proactivity toward controlled regulation, and a resource route, in which comparative evaluation weakens the effectiveness appraisals and recognition that would otherwise offset the cost of proactive investment*.

### Psychological safety

Psychological safety refers to the shared belief that speaking up, taking initiative, or challenging the status quo will not result in punishment or humiliation (Edmondson, 1999). In low psychological safety environments, every proactive episode carries a social risk that prescribed task behavior does not: the employee is acting without instruction, often challenging existing arrangements, and cannot know in advance how colleagues or supervisors will respond. Over repeated episodes, the emotional cost accumulates regardless of outcome; the employee pays it whether the initiative succeeds or fails (Edmondson and Lei, 2014).

Over repeated episodes, social punishment of proactive attempts also produces avoidance schemas in which proactive initiation becomes associated with both interpersonal threat and self-threat simultaneously, and these schemas are particularly resistant to revision because they are affectively charged and identity-relevant (Fiske and Taylor, 1991). Farrell et al. (2025) found that unit-level psychological safety climate mediates the cross-level relationship between relational coordination and individual proactive behaviors, consistent with safety climate operating as a structural enabler of initiative. The present framework extends this by specifying the mechanism: safety shapes not only whether initiative occurs but the emotional cost incurred when it does. Consider two employees who both successfully propose a workflow change. In a high safety climate, the initiator experiences the episode as resource-generative: the initiative succeeded, the ownership claim was validated, and the social risk paid off. In a low safety climate, the same objective outcome is experienced differently: the employee anticipated punishment, managed anxiety through the proposal, and monitored colleagues' reactions throughout. Even success is exhausting. The depletion difference between these two employees is not attributable to effectiveness, both initiatives worked, but to the chronic emotional tax of proactive risk-bearing under different safety conditions. This is a cross-level direct effect: a unit-level climate property predicting an episode-level outcome within persons.

*Proposition 6: Low psychological safety increases depletion following proactive episodes independently of effectiveness appraisals, because the social risk of taking initiative is incurred at the moment of acting, not determined by the outcome*.

### Employment insecurity and precarity

Job insecurity, the perceived threat of involuntary job loss (Greenhalgh and Rosenblatt, 1984), creates pressure to be proactive that has nothing to do with autonomous motivation: employees who feel insecure are motivated to demonstrate value, signal irreplaceability, and stay visible to decision-makers. This is proactivity enacted from fear, not genuine change orientation, the controlled motivational basis Strauss et al. (2017) identified as the condition under which proactivity produces strain. But fear does something else too: it makes even a successful outcome feel like insufficient proof against the threat of job loss, so the reassurance a good outcome would normally provide gets discounted. Lebel et al. (2023) showed that financial precarity can motivate proactive skill building through fear, but that this fear-driven proactivity was associated with higher burnout over time, particularly among employees with stronger impression management motives. Job insecurity shifts the motivational basis of proactivity toward controlled regulation and may push employees toward declining trajectories regardless of their dispositional proactivity or regulatory capacity.

The combination of job insecurity and competitive climate is particularly damaging. Insecure employees in competitive climates must be proactive to signal value, but the competitive context ensures their efforts are evaluated through zero-sum comparison, which weakens the resource-restorative value of even successful initiative. Röllmann et al. (2021) showed that job insecurity moderated the fatigue consequences of voice: voice increased fatigue when insecurity was high but reduced fatigue when insecurity was low. Future research should examine whether this interaction is additive or synergistic, and whether any organizational practice can buffer it.

The gig economy and platform work are a theoretically important boundary condition. Gig workers typically face chronic insecurity, minimal organizational support, and performance systems that are explicitly competitive and outcome-focused. The framework predicts these conditions will produce mostly controlled proactivity and a higher risk of decline. Testing proactivity trajectories in non-standard employment would show whether the conditions identified here work differently outside conventional employment relationships.

*Proposition 7: Job insecurity will predict more proactive behavior in the short term but greater depletion and decline over time, through a motivational route (fear shifting proactivity toward controlled regulation) and a resource route (the threat of job loss discounting even effective outcomes as insufficient reassurance)*.

### Performance management and leadership behavior

The four conditions described above shape the structure within which proactivity unfolds. Performance management and leadership behavior provide 2 day-to-day channels through which that structure is experienced.

Performance management shapes whether employees receive accurate signals about the value of their proactive efforts. Feedback that focuses on outcomes is particularly problematic for proactivity, because proactive initiatives frequently take time to produce measurable results (Grant et al., 2009). An employee whose proposal is under review, or whose process change has not yet been implemented, receives no positive signal from outcome-focused feedback, not because the initiative was poor, but because the feedback cycle is shorter than the proactivity cycle. Contribution-focused feedback is not blanket praise for effort. It judges the reasoning behind an initiative, its usefulness, and what was learned, before final outcomes can be observed. Proactive initiatives often pay off late or uncertainly, so this kind of feedback returns resources during exactly the period in which outcome-focused systems return nothing.

*Proposition 8: Employees who receive contribution-focused feedback will show less decline of proactive confidence over time than those who receive outcome-focused feedback, independently of whether their initiatives actually succeeded*.

Leaders matter because they shape how many organizational conditions are experienced locally. A policy can say initiative is valued; whether it actually feels safe and worthwhile is something an employee learns from how their leader responds to a specific attempt. Two of the mechanisms already described in this paper run largely through the leader: feedback timing and framing and the local enactment of psychological safety both happen leader by leader, not as abstractions. What leaders add beyond passing along feedback and shaping local safety is advocacy: making a contribution visible to others, defending a reasonable failure, and ensuring credit reaches the person who did the work. Political skill and social capital shape how proactive contributions get evaluated (Wihler et al., 2017), and a leader with both can protect an employee's proactive investment from being read as a zero-sum loss elsewhere in the organization.

*Proposition 9: Recognition and political-visibility conditions will predict sustainable rather than declining or suppressed proactivity trajectories through leader advocacy, leaders making initiative visible and defending reasonable failure*.

### Cross-level effects

Individual self-regulatory capacity, autonomous motivation, and dispositional proactivity all shape whether initiative is sustained. However, these individual resources operate within limits set by the organization. When these limits are sufficiently constraining, individual capacity cannot compensate. Under chronically depleting conditions, even high-capacity, autonomously motivated employees will follow declining trajectories. The logic follows from two premises established above. First, organizational conditions that frustrate basic psychological needs deplete motivational resources (Vansteenkiste and Ryan, 2013). Second, psychological ownership means that proactive investment in hostile organizational contexts generates amplified loss when initiatives are unsupported or unrecognized, accelerating depletion beyond what effectiveness appraisals alone would predict (Pierce et al., 2003). Chronic structural hostility can override individual resources across months, not just days. The ceiling is not entirely fixed, and the conditions differ in how far the behavior they bound can reshape them. Work design and an immediate leader's responses are comparatively open to it: Zacher et al. (2019) found that increases in personal initiative predicted later increases in job autonomy, which is the ceiling being raised by the behavior beneath it. Formal reward systems are far less open to any single employee. Unit climates sit in between, ordinarily changing through repeated behavior across many members rather than one person alone. The same conditions that impose a ceiling when hostile can therefore serve as a structural foundation for sustainable proactivity when supportive, and part of that foundation can be laid by proactivity itself.

The same logic could be scaled up: when many employees in a unit sustain proactivity, their combined behavior may gradually build a unit climate in which initiative is expected and supported. Some of the framework's own conditions would then sit partly downstream of the behavior it models. The present specification is one-directional and does not formalize this; it is named as a boundary of the model, and the research agenda notes the designs that could test it.

This argument has a direct implication for how organizations approach proactivity. The literature's antecedent-focused tradition has generated a primarily selection and development logic: hire proactively disposed individuals, build self-efficacy, and enhance motivational states. The sustainability framework implies that this logic is necessary but insufficient. Selection and development raise the individual resource base; organizational design influences the rate at which that base is consumed or replenished. Sustainable proactivity requires both, but when organizations attend only to the former, they expend resources selecting and developing proactive employees only to deploy them into structural conditions that systematically undermine the sustainability of the very behavior they were selected for.

Individual differences in self-regulatory capacity and motivational quality interact with the structural conditions proposed here, a point extensively theorized and empirically supported (Parker et al., 2010; Strauss and Parker, 2014; Cangiano and Parker, 2016). The present contribution foregrounds the organizational conditions that set the ceiling within which individual resources operate. The implication is that individual-level interventions produce gains that decay when structural conditions remain hostile, making the sequencing of intervention critical.

*Proposition 10: When organizational conditions are chronically resource-depleting, structural interventions will produce more durable improvements in sustained proactivity than individual-level interventions alone*.

### AI-augmented and non-standard work

The rapid integration of artificial intelligence into organizational processes creates conditions that simultaneously intensify the demand for proactivity and undermine the psychological conditions under which it can be sustained. A recent multilevel review situates this integration as touching individual, team, and organizational levels simultaneously, consistent with the present framework's own multilevel argument (Bankins et al., 2024). Two mechanisms are particularly relevant for the sustainability framework.

The first concerns motivational displacement. As AI systems increasingly perform routine anticipatory and problem-identification functions, the proactive initiatives remaining for human employees shift toward those requiring relational navigation, ethical judgment, and contextual sensitivity. These are also the most emotionally costly forms of proactivity: they carry the highest social risk, the greatest uncertainty about effectiveness, and the least clear feedback. AI integration may therefore shift the human proactivity domain toward precisely the episodes most likely to produce depletion and most dependent on psychological safety for their sustainability. Attribution ambiguity, below, yields a specific individual-level prediction. Motivational displacement does not; it describes a broad shift in what human proactive work consists of, so it is carried into the research agenda rather than formalized as a proposition.

The second concerns attribution ambiguity. When AI systems contribute to identifying problems or generating solutions, the causal attribution of proactive success becomes uncertain. Causal attribution of success to one's own initiative is central to the mastery experiences that make proactivity resource-generative (Parker, 1998; Wu et al., 2018). Systematic attribution ambiguity will attenuate effectiveness appraisals even when proactive efforts succeed, accelerating appraisal recalibration and pushing trajectories toward contingent or declining patterns. Organizations that develop explicit norms for recognizing human initiative in AI-assisted work will sustain proactivity trajectories more effectively than those that leave attribution implicit.

*Proposition 11: AI attribution ambiguity will weaken the positive effect of proactive success on proactive confidence when employees perceive successful outcomes as attributable primarily to AI rather than to their own initiative*.

Future research should examine how AI integration moderates each of the organizational sustainability conditions identified in the framework. Does AI augmentation of routine tasks increase or decrease effectiveness appraisal probability for residual human proactive initiatives? Does it alter the competitive climate dynamic by changing what counts as distinctive contribution? Does it interact with psychological safety in determining whether human proactivity is experienced as meaningful or superfluous? These questions connect the sustainability framework to the most significant organizational transformation of the present decade.

## Discussion

This paper shifts the question from what makes employees proactive to what allows proactivity to remain sustainable over time. The multilevel framework developed here argues that this question cannot be answered at the individual level alone: organizational conditions set the ceiling within which proactive behavior either compounds or wears down across episodes. Organizations that simultaneously demand proactivity and impose the conditions that make it unsustainable are, in effect, actively generating the conditions for its decline.

### Theoretical implications

#### Proactivity as a trajectory-level phenomenon

Proactivity has been studied almost exclusively as a cross-sectional or short-window within-person phenomenon (Ohly et al., 2010). Recent research has begun to recognize that the quality and manner of proactive behavior matter alongside its frequency (Wang et al., 2025). The present framework extends this shift by arguing that the sustainability of proactive behavior depends on how repeated proactive episodes accumulate under different organizational conditions. This carries theoretical implications beyond the proactivity literature: any episode-level finding about resource dynamics, depletion, or motivation quality may have different meaning depending on where in a trajectory arc it is measured. An episode of controlled proactivity in an early-stage declining trajectory looks identical at the moment of measurement to an episode in a long-established one, but their antecedents, resource implications, and organizational determinants differ substantially. The trajectory level of analysis therefore adds something diary studies cannot capture on their own: what an episode means depends on where it falls in that longer arc. The framework may also inform research on other discretionary, resource-intensive behaviors, including voice, creativity, and organizational citizenship (Bindl and Parker, 2011; Parker and Collins, 2010), but these extensions require careful attention to construct-specific differences and are taken up under future research.

#### Organizational conditions as structural ceilings

Individual differences in autonomous motivation, self-efficacy, and regulatory capacity remain relevant: they shape the initial resource base from which proactive behavior is launched (Parker et al., 2010; Strauss and Parker, 2014; Cangiano and Parker, 2016). The framework argues, however, that organizational conditions shape the rate at which that base is consumed or replenished (Hobfoll et al., 2018), and that chronic structural hostility can override individual resources across months. The same conditions operate in both directions: ceilings when hostile, a structural foundation for sustainable proactivity when supportive. This extends the contingency account developed across recent proactivity research (Strauss et al., 2017; Wehrt and Sonnentag, 2024; Cangiano et al., 2026) from the episode level to the trajectory level, and from individual contingencies to organizational structural conditions. It also identifies psychological ownership over the work role and change domain (Pierce et al., 2001, 2003) and need frustration (Vansteenkiste and Ryan, 2013) as the mechanisms explaining why organizational conditions produce stronger and more durable effects on proactive than on prescribed task behavior. Competitive climate has been treated primarily as a context variable that moderates individual-level outcomes (Fletcher et al., 2008; Cangiano et al., 2026). Psychological safety has been extensively studied as an antecedent of voice and learning behavior (Edmondson, 1999; Edmondson and Lei, 2014). The present argument, that both accumulate their effects over time as opposed to operating only episode by episode, implies that their full impact cannot be seen in cross-sectional designs. Capturing their full organizational significance requires longitudinal examination. A further question, the framework surfaces but does not resolve is whose sustainability is the relevant criterion. Coding the contingent trajectory as organizationally suboptimal, output capped by fluctuating resource availability, privileges the organization's interest in sustained initiative; from the employee's perspective, the same self-regulation may represent adaptive self-protection rather than a shortfall.

#### From declining proactivity to burnout

The declining trajectory described here suggests a specific pathway from proactivity to burnout that has not previously been theorized: repeated controlled-motivation proactive episodes progressively deplete resources through motivational drift, eventually producing the exhaustion and disengagement that characterize burnout (Demerouti et al., 2001). The controlled motivational basis, which Strauss et al. (2017) identified as the primary condition under which proactivity produces strain, becomes self-sustaining as resources run down: employees continue initiating because structural or insecurity pressures demand it, while each episode generates less resource return than the last. This pathway is distinct from generic demand overload, where exhaustion follows from high prescribed demands. In the declining trajectory, burnout emerges precisely because employees are motivated enough to keep initiating while structural conditions ensure those initiatives generate insufficient resource return, and because organizations actively promote the behavior that drives the depletion. The framework does not establish this pathway empirically; it identifies it as a testable proposition with implications for how organizations should interpret burnout among their most initiative-oriented employees.

### Practical implications

Organizations routinely invest in selecting proactive employees and building their motivational capacity without attending to the conditions that shape whether that investment holds or erodes over time. Sustainable proactivity requires not only proactively disposed employees but organizations designed to replenish the resources that proactive behavior consumes.

This reorients the questions practitioners should be asking. The diagnostic is not primarily whether employees are sufficiently motivated or skilled to take initiative, but whether organizational conditions make proactive success reliably achievable. Are proactive efforts evaluated on contribution quality, or only on whether they produced a measurable outcome within the appraisal cycle, given that the feedback cycle is typically shorter than the proactivity cycle (Grant et al., 2009)? Are proactive initiatives recognized individually, or do they depend on performance comparisons that convert initiative into zero-sum competition? Do employees have enough autonomy to select proactive targets that are feasible as opposed to socially risky? Is initiative safe when it fails, or only when it produces visible results? Do leaders actively make employees' contributions visible and defend reasonable failures, or leave initiative to speak for itself? And are employees under job insecurity performing initiative as a visibility signal rather than from genuine change motivation?

Of these conditions, reward system reform and psychological safety development are likely to produce the largest trajectory-level effects, because they act on multiple depleting mechanisms at once and are the conditions most commonly misaligned with proactivity sustainability in contemporary high-performance work systems. Organizations that invest heavily in proactivity training and selection while maintaining competitive reward structures and low safety climates are, in effect, selecting for the declining trajectory: recruiting motivated initiators and deploying them into structural conditions that will systematically exhaust that motivation over time.

### Limitations

Several limitations should be acknowledged. The four-trajectory typology is theoretical; empirical validation using longitudinal designs is required before it can be treated as more than a heuristic organizing framework. The typology also assigns one trajectory per employee. As noted in the introduction, behaviors inside the defined domain can move independently, and the framework does not model them separately. An employee whose voice declines while taking charge holds steady would be assigned a single trajectory that describes neither well. Whether the behaviors within the domain converge over time, and what conditions pull them apart, is a question for the parallel-process designs described below. The boundary conditions of the organizational propositions may vary by context: the effects of competitive climate may differ between individualistic and collectivistic cultures, between knowledge work and operational roles, and between contexts with high vs. low task interdependence. The competitive-climate finding central to Proposition 5 also currently rests in part on a single source not yet in final publication (Cangiano et al., 2026); the broader argument is not intended to depend on this result alone. Nor does the framework's treatment of competition and contingent reward as depleting rule out boundary conditions: trait competitiveness and the distinction between informational and controlling administration of rewards may both attenuate the effect documented here (Fletcher et al., 2008). The framework also treats organizational conditions as relatively stable, but organizations change, and abrupt shifts in reward structure, leadership, or climate may produce trajectory transitions that the present model does not fully account for. Finally, the framework focuses on individual proactivity. The theoretical architecture developed here centers on how individual employees navigate organizational conditions over time, and does not theorize collective proactivity, team-level voice systems, or the institutional mechanisms through which initiative is channeled and evaluated collectively. Extending the framework to these levels would require different mechanisms and different empirical designs, and readers should not infer that the present argument applies straightforwardly to collective voice or team initiative.

### Future research

The most pressing methodological need is longitudinal density. The field's reliance on 5-day diary designs has produced a literature rich in episode-level findings but structurally unable to observe trajectory-level dynamics (Ohly et al., 2010). Testing the trajectory propositions requires intensive longitudinal designs spanning a minimum of 8–12 weeks. Eight to twelve weeks may be enough to tell emerging trajectory classes apart. Catching transitions between classes will likely take much longer observation, and the latent transition analyses below assume those longer horizons. Because a trajectory is defined here on two processes at once, measurement has to cover both. Studies should track the frequency of change-oriented initiative and, on the same schedule, role breadth self-efficacy and the autonomous or controlled quality of the motivation behind that initiative. Modeling the two as parallel processes, through a parallel-process latent growth model, allows the declining class to be identified by the divergence between them. Estimating both pathways in one model as parallel mediators also tests Proposition 3 directly. The distinguishing pattern is autonomous motivation holding while self-efficacy falls and initiative later follows, under chronically attenuated appraisals; a need-quality account does not predict it. Frequency alone cannot recover that class, since declining employees continue to initiate at close to their previous rate. Proposition 1 predicts trajectories from episode-level resource balance, so the two cannot come from the same measurement. Studies should measure resource balance directly, with its own items, rather than reading it off the trajectory an employee follows; otherwise the test is circular. Group-based trajectory modeling (Nagin, 2005) applied to the joint pattern would allow empirical identification of trajectory clusters. The test the framework implies is a cross-level interaction: individual resources should predict trajectory membership and change only where conditions permit, and studies should model that interaction directly. Unit-level climates such as psychological safety and competitive climate should be operationalized from aggregated member perceptions, with within-unit agreement established before aggregation. Cross-lagged or similar reciprocal designs could then test whether sustained proactivity reshapes work conditions and, in the aggregate, feeds a unit-level climate, rather than leaving that assumed. Latent transition analysis would additionally allow tests of when employees move between trajectories, and whether movement out of the declining class occurs at all.

Cross-organizational panel designs are needed to test the organizational propositions. Sufficient variance in work design, reward systems, competitive climate, and psychological safety is unlikely within a single organization. Multi-organization designs analogous to those used in burnout research (Demerouti et al., 2001) would allow direct cross-level tests of whether structural conditions predict trajectory type independently of individual characteristics. Including organizations at different points on the competitive-collaborative reward continuum would be particularly informative for testing Proposition 5. Natural experiments offer a compelling design for testing trajectory transitions. Organizations that shift reward structures, implement new performance management systems, or undergo significant leadership change provide exogenous variation in the conditions hypothesized to shape trajectory type. Measuring proactivity trajectories before and after such changes, with at least 6 months of post-change follow-up, would allow direct tests of whether structural organizational changes shift employees toward more sustainable proactivity patterns. Intervention studies comparing individual-level conditions (self-efficacy building, autonomy restoration, and mastery experience) against structural redesign conditions would test Proposition 10 directly. The prediction is that individual-level interventions produce gains that decay in the absence of structural support, a practically significant claim with direct implications for how organizations allocate resources between development programs and structural reform. Finally, future research should examine whether the trajectory logic transfers to other discretionary, resource-intensive behaviors. Creativity and organizational citizenship behavior share proactivity's key structural features: they are self-initiated, resource-consuming, and dependent on perceived effectiveness for their resource-generative effects (Bindl and Parker, 2011; Parker and Collins, 2010). However, these behaviors differ in their social risk profiles, recognition dynamics, and feedback availability, and the mechanisms theorized here cannot be assumed to transfer without construct-level analysis.

## Conclusion

Proactive work behavior is not simply a function of individual motivation or disposition. It is a behavioral pattern that unfolds over time in an organizational context that either creates or forecloses the conditions for its sustainability. Most organizations demand proactivity while structuring the conditions that make it unsustainable. The present framework makes this contradiction explicit, identifies its organizational mechanisms, and derives a research agenda for testing it. The management of proactivity requires organizational design alongside individual development, and attending to that distinction is the practical challenge the framework is intended to address.

## Data Availability

The original contributions presented in the study are included in the article/supplementary material, further inquiries can be directed to the corresponding author.

## References

[B1] Adams J. S. (1965). “Inequity in social exchange,” in Advances in Experimental Social Psychology, Vol. 2, ed. L. Berkowitz (New York, NY: Academic Press), 267–299.

[B2] Appelbaum E. Bailey T. Berg P. Kalleberg A. L. (2000). Manufacturing Advantage: Why High-Performance Work Systems Pay Off . Ithaca, NY: Cornell University Press.

[B3] Bankins S. Ocampo A. C. Marrone M. Restubog S. L. D. Woo S. E. (2024). A multilevel review of artificial intelligence in organizations: implications for organizational behavior research and practice. J. Organ. Behav. 45, 159–182. doi: 10.1002/job.2735

[B4] Bindl U. K. Parker S. K. (2011). “Proactive work behavior: forward-thinking and change-oriented action in organizations,” in APA Handbook of Industrial and Organizational Psychology, Vol. 2, ed. S. Zedeck (Washington, DC: American Psychological Association), 567–598. doi: 10.1037/12170-019

[B5] Bindl U. K. Parker S. K. Totterdell P. Hagger-Johnson G. (2012). Fuel of the self-starter: how mood relates to proactive goal regulation. J. Appl. Psychol. 97, 134–150. doi: 10.1037/a0024368 21744938

[B6] Bolino M. C. Valcea S. Harvey J. (2010). Employee, manage thyself: the potentially negative implications of expecting employees to behave proactively. J. Occup. Organ. Psychol. 83, 325–345. doi: 10.1348/096317910X493134

[B7] Cangiano F. Parker S. K. (2016). “Proactivity for mental health and well-being,” in The Wiley Blackwell Handbook of the Psychology of Occupational Safety and Workplace Health, ed. S. Clarke, T. M. Probst, F. Guldenmund, and J. Passmore (Chicester: John Wiley and Sons), 228–250. doi: 10.1002/9781118979013.ch11

[B8] Cangiano F. Parker S. K. Ouyang K. (2021). Too proactive to switch off: when taking charge drains resources and impairs detachment. J. Occup. Health Psychol. 26, 142–154. doi: 10.1037/ocp0000265 33104372

[B9] Cangiano F. Parker S. K. Yeo G. B. (2019). Does daily proactivity affect well-being? The moderating role of punitive supervision. J. Organ. Behav. 40, 59–72. doi: 10.1002/job.2321

[B10] Cangiano F. To M. L. Sander L. (2026). Proactivity effectiveness alleviates the psychological costs of proactive behavior in low competitive climates. Aus. J. Manag. doi: 10.1177/03128962261470853

[B11] Crant J. M. (2000). Proactive behavior in organizations. J. Manag. 26, 435–462. doi: 10.1177/014920630002600304

[B12] Demerouti E. Bakker A. B. Nachreiner F. Schaufeli W. B. (2001). The job demands–resources model of burnout. J. Appl. Psychol. 86, 499–512. doi: 10.1037/0021-9010.86.3.499 11419809

[B13] Edmondson A. C. (1999). Psychological safety and learning behavior in work teams. Adm. Sci. Q. 44, 350–383. doi: 10.2307/2666999

[B14] Edmondson A. C. Lei Z. (2014). Psychological safety: the history, renaissance, and future of an interpersonal construct. Annu. Rev. Organ. Psychol. Organ. Behav. 1, 23–43. doi: 10.1146/annurev-orgpsych-031413-091305

[B15] Farrell J. B. Flood P. C. Hodgkinson G. P. Kilroy S. Rivkin W. Strauss K. (2025). The impact of positive work relationships on proactive behaviors: a multilevel study. Appl. Psychol. 74:e70029. doi: 10.1111/apps.70029

[B16] Fay D. Hüttges A. (2017). Drawbacks of proactivity: effects of daily proactivity on daily salivary cortisol and subjective well-being. J. Occup. Health Psychol. 22, 429–442. doi: 10.1037/ocp0000042 27182764

[B17] Fiske S. T. Taylor S. E. (1991). Social Cognition, 2nd Edn. New York, NY: McGraw-Hill.

[B18] Fletcher T. D. Major D. A. Davis D. D. (2008). The interactive relationship of competitive climate and trait competitiveness with workplace attitudes, stress, and performance. J. Organ. Behav. 29, 899–922. doi: 10.1002/job.503

[B19] Frese M. Fay D. (2001). Personal initiative: an active performance concept for work in the 21st century. Res. Organ. Behav. 23, 133–187. doi: 10.1016/S0191-3085(01)23005-6

[B20] Grant A. M. Ashford S. J. (2008). The dynamics of proactivity at work. Res. Organ. Behav. 28, 3–34. doi: 10.1016/j.riob.2008.04.002

[B21] Grant A. M. Parker S. K. Collins C. G. (2009). Getting credit for proactive behavior: supervisor reactions depend on what you value and how you feel. Pers. Psychol. 62, 31–55. doi: 10.1111/j.1744-6570.2008.01128.x

[B22] Greenhalgh L. Rosenblatt Z. (1984). Job insecurity: toward conceptual clarity. Acad. Manag. Rev. 9, 438–448. doi: 10.2307/258284

[B23] Halbesleben J. R. Neveu J.-P. Paustian-Underdahl S. C. Wheeler M. (2014). Getting to the “COR”: understanding the role of resources in conservation of resources theory. J. Manage. 40, 1334–1364. doi: 10.1177/0149206314527130

[B24] Hobfoll S. E. (1989). Conservation of resources: a new attempt at conceptualizing stress. Am. Psychol. 44, 513–524. doi: 10.1037/0003-066X.44.3.513 2648906

[B25] Hobfoll S. E. Halbesleben J. Neveu J.-P. Westman M. (2018). Conservation of resources in the organizational context: the reality of resources and their consequences. Annu. Rev. Organ. Psychol. Organ. Behav. 5, 103–128. doi: 10.1146/annurev-orgpsych-032117-104640

[B26] Hobfoll S. E. Shirom A. (2001). “Conservation of resources theory: applications to stress and management in the workplace,” in Handbook of Organizational Behavior, ed. R. T. Golembiewski (NewYork, NY: Marcel Dekker), 57–80.

[B27] Hyatt D. E. Ruddy T. M. (1997). An examination of the relationship between work group characteristics and performance. Pers. Psychol. 50, 553–585. doi: 10.1111/j.1744-6570.1997.tb00703.x

[B28] Lebel R. D. Yang X. Parker S. K. Kamran-Morley D. (2023). What makes you proactive can burn you out: the downside of proactive skill building motivated by financial precarity and fear. J. Appl. Psychol. 108, 1207–1222. doi: 10.1037/apl0001063 36455018

[B29] Mäkikangas A. Kinnunen U. (2016). The person-oriented approach to burnout: a systematic review. Burn. Res. 3, 11–23. doi: 10.1016/j.burn.2015.12.002

[B30] Nagin D. S. (2005). Group-based Modeling of Development. Cambridge, MA: Harvard University Press. doi: 10.4159/9780674041318

[B31] Ohly S. Sonnentag S. Niessen C. Zapf D. (2010). Diary studies in organizational research: an introduction and some practical recommendations. J. Pers. Psychol. 9, 79–93. doi: 10.1027/1866-5888/a000009

[B32] Parker S. K. (1998). Enhancing role breadth self-efficacy: the roles of job enrichment and other organizational interventions. J. Appl. Psychol. 83, 835–852. doi: 10.1037/0021-9010.83.6.835 9885197

[B33] Parker S. K. Bindl U. K. Strauss K. (2010). Making things happen: a model of proactive motivation. J. Manage. 36, 827–856. doi: 10.1177/0149206310363732

[B34] Parker S. K. Collins C. G. (2010). Taking stock: integrating and differentiating multiple proactive behaviors. J. Manage. 36, 633–662. doi: 10.1177/0149206308321554

[B35] Parker S. K. Williams H. M. Turner N. (2006). Modeling the antecedents of proactive behavior at work. J. Appl. Psychol. 91, 636–652. doi: 10.1037/0021-9010.91.3.636 16737360

[B36] Pierce J. L. Kostova T. Dirks K. T. (2001). Toward a theory of psychological ownership in organizations. Acad. Manag. Rev. 26, 298–310. doi: 10.2307/259124

[B37] Pierce J. L. Kostova T. Dirks K. T. (2003). The state of psychological ownership: integrating and extending a century of research. Rev. Gen. Psychol. 7, 84–107. doi: 10.1037/1089-2680.7.1.84

[B38] Pingel R. Fay D. Urbach T. (2019). A resources perspective on when and how proactive work behaviour leads to employee withdrawal. J. Occup. Organ. Psychol. 92, 410–435. doi: 10.1111/joop.12254

[B39] Röllmann L. F. Weiss M. Zacher H. (2021). Does voice benefit or harm occupational well-being? The role of job insecurity. Br. J. Manag. 32, 708–724. doi: 10.1111/1467-8551.12471

[B40] Ryan R. M. Deci E. L. (2000). Self-determination theory and the facilitation of intrinsic motivation, social development, and well-being. Am. Psychol. 55, 68–78. doi: 10.1037/0003-066X.55.1.68 11392867

[B41] Sonnentag S. (2003). Recovery, work engagement, and proactive behavior: a new look at the interface between nonwork and work. J. Appl. Psychol. 88, 518–528. doi: 10.1037/0021-9010.88.3.518 12814299

[B42] Strauss K. Parker S. K. (2014). “Effective and sustained proactivity in the workplace: a self-determination theory perspective,” in The Oxford Handbook of Work Engagement, Motivation, and Self-Determination Theory, ed. M. Gagné (Oxford: Oxford University Press), 50–71.

[B43] Strauss K. Parker S. K. O'Shea D. (2017). When does proactivity have a cost? Motivation at work moderates the effects of proactive work behavior on employee job strain. J. Vocat. Behav. 100, 15–26. doi: 10.1016/j.jvb.2017.02.001

[B44] Tims M. Bakker A. B. Derks D. (2012). Development and validation of the job crafting scale. J. Vocat. Behav. 80, 173–186. doi: 10.1016/j.jvb.2011.05.009

[B45] Vansteenkiste M. Ryan R. M. (2013). On psychological growth and vulnerability: basic psychological need satisfaction and need frustration as a unifying principle. J. Psychother. Integr. 23, 263–280. doi: 10.1037/a0032359

[B46] Wang Y. Parker S. K. Wu C. H. Zhou M. Liao J. (2025). Wise proactivity: antecedents, outcomes, and a mechanism. J. Organ. Behav. 46, 923–938. doi: 10.1002/job.2887

[B47] Wehrt W. Sonnentag S. (2024). When is taking charge depleting? Job control and self-control demands as moderators in daily depletion processes. Scand. J. Work Organ. Psychol. 9, 1–17. doi: 10.16993/sjwop.219

[B48] Wihler A. Blickle G. Ellen B. P. Hochwarter W. A. Ferris G. R. (2017). Personal initiative and job performance evaluations: role of political skill in opportunity recognition and capitalization. J. Manage. 43, 1388–1420. doi: 10.1177/0149206314552451

[B49] Wu C. H. Deng H. Li Y. (2018). Enhancing a sense of competence at work by engaging in proactive behavior: the role of proactive personality. J. Happiness Stud. 19, 801–816. doi: 10.1007/s10902-016-9827-9

[B50] Xu S. Jia J. Wang Y. Shah P. P. Xie L. Wu T. (2026). Fueling employees' initiative: a meta-analysis of the motivational antecedents of proactive work behavior. J. Organ. Behav. 47, 399–419. doi: 10.1002/job.70018

[B51] Zacher H. Schmitt A. Jimmieson N. L. Rudolph C. W. (2019). Dynamic effects of personal initiative on engagement and exhaustion: the role of mood, autonomy, and support. J. Organ. Behav. 40, 38–58. doi: 10.1002/job.2277

